# Recognition of Non-Harmonic Natural Sounds by Small Mammals Using Competitive Training

**DOI:** 10.1371/journal.pone.0051318

**Published:** 2012-12-10

**Authors:** Hisayuki Ojima, Masato Taira, Michinori Kubota, Junsei Horikawa

**Affiliations:** 1 The Center for Brain Integration Research (CBIR) and Cognitive Neurobiology, Graduate School of Medical and Dental Sciences, Tokyo Medical and Dental University, Tokyo, Japan; 2 Neuroinformatics, Medical Research Institute, Tokyo Medical and Dental University, Tokyo, Japan; 3 Graduate School of Computer Science and Engineering, Toyohashi University of Technology, Aichi, Japan; UNLV, United States of America

## Abstract

Animals recognize biologically relevant sounds, such as the non-harmonic sounds made by some predators, and respond with adaptive behaviors, such as escaping. To clarify which acoustic parameters are used for identifying non-harmonic, noise-like, broadband sounds, guinea pigs were conditioned to a natural target sound by introducing a novel training procedure in which 2 or 3 guinea pigs in a group competed for food. A set of distinct behavioral reactions was reliably induced almost exclusively to the target sound in a 2-week operant training. When fully conditioned, individual animals were separately tested for recognition of a set of target-like sounds that had been modified from the target sound, with spectral ranges eliminated or with fine or coarse temporal structures altered. The results show that guinea pigs are able to identify the noise-like non-harmonic natural sounds by relying on gross spectral compositions and/or fine temporal structures, just as birds are thought to do in the recognition of harmonic birdsongs. These findings are discussed with regard to similarities and dissimilarities to harmonic sound recognition. The results suggest that similar but not identical processing that requires different time scales might be used to recognize harmonic and non-harmonic sounds, at least in small mammals.

## Introduction

Animal habitats are rich in environmental sounds. Environmental natural sounds can be classified into two global groups based on their spectral structures: harmonic and non-harmonic sounds [1]. Human vocalizations, animal calls, and birdsongs are generally harmonic in structure, last a relatively long time, and are used frequently for conspecific communication [2]-[5]. Conversely, ambient sounds that animals are exposed to in their environments are generally non-harmonic and of short duration. Some of these sounds may gain biological significance, most likely as a consequence of exposure or learning. For example, rustling sounds produced by moving prey provide binaural cues that barn owls exploit to localize their prey [6]. In other situations, non-harmonic, noise-like sounds produced by movements of approaching predators can induce adaptive responses, such as escaping or freezing, in the listening prey. Similarly, animals in captivity can anticipate food or water from various sounds made by animal keepers approaching the cage, and these sounds may become attractive signals to these animals.

Natural sounds are complex in both spectral and temporal dimensions [7]–[8]. In contrast to harmonic calls or songs, ambient sounds generally take the form of non-harmonic, broadband noise with time-varying amplitude envelopes or multiple local spectral peaks of energy [1]. Differences in spectral composition and biological significance between harmonic and non-harmonic sounds are assumed to reflect distinctive neural mechanisms that encode these sounds. For example, it is postulated that harmonic and non-harmonic sounds are differently decoded, even in the cochlea. Sounds such as animal communication calls may be filtered largely on the basis of Fourier transformations with flat spectral filter properties for a relatively long time, while environmental sounds are filtered on the basis of wavelet transformations with peaked filter properties that are effective for a short time. Human speech sounds share both decoding features because they have both harmonic vowels and non-harmonic consonants [9]. However, higher-order processing of these differentially decoded sounds is still unclear.

In contrast to the recognition mechanisms described for the harmonic songs of birds [10]-[12], the recognition mechanisms of small mammals for ambient noise-like sounds have not been fully studied. Nonetheless, it is known that ambient sounds can transmit information that invokes adaptive behaviors in receivers. If animals detect approaching objects, they are preferentially attentive to auditory and/or visual cues of the objects [13]–[14], and they display escape or approach behavior, depending on the current context or past experience. This biologically important adaptive behavior demands the auditory processing of non-harmonic sounds together with the accessing of brain regions related to decision-making and motor output [8].

While caring for guinea pigs, we noticed that they showed adaptive behaviors in response to ambient noises that were generated during feeding procedures. Animals frequently emitted calls [15] and initiated distinct actions whenever an animal keeper started to feed them. Guinea pigs have been extensively used for studies of auditory periphery [16], but they have rarely been used for behavioral studies using non-aversive stimuli. This may be because guinea pigs are difficult to train stably in isolation and are highly sensitive to unfamiliar stimuli. Despite these difficulties in training, it is evident that the daily feeding procedure easily and consistently evokes stereotypic responses to a non-harmonic sound. This observation motivated us to examine which acoustic parameters were used for food anticipation. To address this question, we adopted a noise-like natural sound (the keeper’s footstep) as the conditioning stimulus and evaluated which acoustic parameters were being used as recognition cues. Noise-like sounds have been evaluated generally as parts of more complicated sounds, such as plosives and fricatives of human consonants, or as infrequent insertions within a birdsong motif of harmonically structured syllables [12]. Because the footstep sound is noise-like for its entire length, we first modified the entire sound by gross elimination of wide ranges of spectral components, changes to the overall envelope configuration, and disturbance to the timing of individual segments of the multi-segment sound.

We assumed that the guinea pigs would exhibit the stereotypic behavioral reactions to the conditioned sound consistently if they perceived a given modified sound to be the same as, or in the same category as, the conditioned sound. Conversely, they would not display these reactions if they recognized a modified sound as different from the conditioned sound; instead, they would react in the same way as they do to a distracting sound. Alternatively, they would refrain from responding if they perceived a modified sound to be novel, just as they behaved in reaction to unknown sounds before training.

## Materials and Methods

### Animals and Training Facility

The care and use of animals in this experiment were approved by the animal committee of the Tokyo Medical and Dental University (no. 0120046B and no. 0130268A) and conformed to the National Institutes of Health Guide for the Care and Use of Laboratory Animals (NIH publications No. 80–23, revised in 1996).

Guinea pigs (Hartley, male, body weight of approximately 250 g) purchased from a commercial supplier (Japan SLC) were initially kept in the university animal facility under a free-feeding condition at a standard light-dark cycle (lights on at 8∶00 and off at 18∶00). When animals reached 400–500 g in weight, they were transferred to our laboratory (temperature set at 22–23°C). Two or 3 guinea pigs were housed together in a single cage under the same light-dark cycle as the animal facility.

Training was carried out in an arena placed inside a sound-attenuated chamber lined with urethane form. The training arena (W50×D50×H30 cm) was made of metal-mesh walls on all sides and a sound-absorbing carpet on the floor. A custom-made pellet dispenser connected to a food hopper was set on one wall. Pellets were fed into a saucer (10 cm in diameter) through the hopper. To monitor animal behaviors and sound delivery [17], we placed 1 microphone (F-720, SONY) 60 cm above the food saucer, 3 video cameras (WAT-204CX, Watec, Japan and SH-6C, WTW, Japan) at 3 corners of the arena, and a custom-made motion detector 45 cm above the food saucer to cover its 10 cm diameter. The motion detector was used to determine precisely the timing at which the animal's motion above the food saucer was initiated.

### Sound Delivery System

Two identical loudspeakers (NS-10MM, Yamaha, Japan) were set 1.7 m above the arena and separated 1 m from each other. Stimulus sounds were played back from the loudspeakers via a power amplifier (N220, Sony, Japan) and an analog equalizer (Q2031B, Yamaha, Japan) with a frequency range of 80 Hz to 12 kHz. The sound delivery system was calibrated at 60 cm above the food saucer using a half-inch condenser microphone (type 7012, ACO, Japan). The output of the system was compensated at 25 spectral points (1/3 octave) with the equalizer to make the fluctuation level as small as possible (i.e., ±6 dB at 63 dB SPL).

### Training Stimulus Sounds and their Parameters

**Figure 1:**
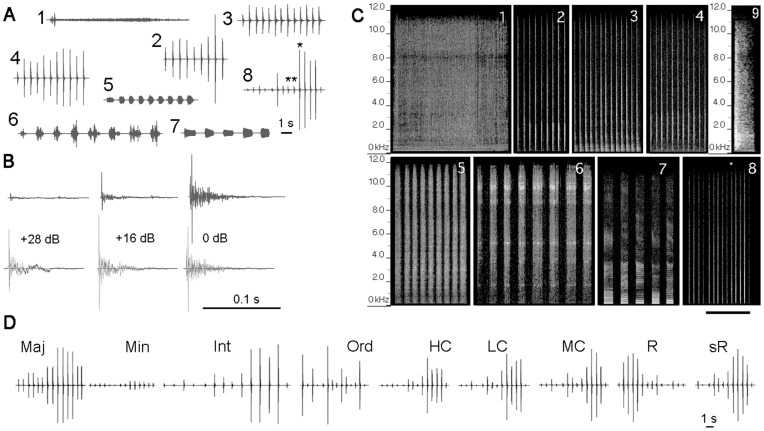
Sound stimuli used for training and the recognition tests. A , Waveforms of the training stimulus set, including non-target (1–7) and target sounds (8). Food is associated only with the target sound. Sound type: 1, tap water flowing in a sink; 2, clapping hands; 3, hitting a plastic carrier; 4, hitting a metal cage; 5, scratching a metal mesh; 6, jingling keys; 7, human vowels; and 8, human footsteps. **B**, Details of the envelope shape of individual segments of the step target sound. In the upper panels, the time (horizontal) axis is expanded for the 8th, 9th and 10th segments (asterisks in **A**–8), while in the lower panels, the amplitude (vertical) axis is enlarged by the value shown in dB. **C**, Spectrograms of the stimulus sounds. The numerals correspond to those shown in **A**. The 10th segment of the step sound (asterisk in c–8), expanded in time, is shown in C–9. The energy level is represented by a gray scale, with white for the highest level and black for the lowest level. Note that all the stimuli except the vowels (7) show non-harmonic noise-like spectra. The calibration is 5 s for all panels except for panel 9 (0.25 s). **D**, Waveforms of the stimulus set used for recognition tests. “Maj” and “Min”; modified step sounds with 7 major and minor segments of the step sound doubled. “Int”; modified step sound with the intersegment intervals expanded. “Ord”; modified step sound with segment-order shuffled. “LC”, “MC” and “HC”; modified step sounds with the low-, mid-, and high-frequency ranges removed from the step sound, respectively. “R” and “sR”; modified step sounds with the entire sound reversed in time and with individual segments locally time-reversed without changes in their order, respectively.

For the training stimulus set, 1 targer (T) and 7 non-target (NT) sounds were recorded with a condenser microphone (type 7146, ACO, Japan) and digitized at a sampling rate of 44.1 kHz and 16-bit length with sound editing software (Amadeus Pro, HairerSoft, UK) in a Macintosh platform computer. The footstep sound (T sound) was recorded while a feeder was actually walking on the laboratory floor (W 4.3×D 6.6×H 2.7 m). Various types of NT natural sounds, to which animals had been exposed frequently, occasionally or rarely, were collected at a distance of 1.0 m from the microphone. The frequent sounds included tap water running in a sink below the cage, hitting a cage on a metal plate, and scratching the metal mesh of a cage; the occasional sounds included hitting a plastic carrier and pronouncing Japanese vowels (by a Japanese male); and the rare sounds included clapping hands and jingling keys (Fig. 1A).

The step sound consisted of 14 segments of varying amplitudes but of a relatively similar envelope shape (Fig. 1B), with the segment length ranging from 74 to 142 ms (110±19). The amplitude envelopes were asymmetric in time, with gradually damping tails. Each segment displayed a spectral composition similar to a broadband noise (ranging from 0.10 to 12.0 kHz, Fig. 1C), with dominant local energy peaks at approximately 0.6 kHz, 1.0 kHz, 1.3 kHz, 1.6 kHz, 2.5 kHz, and 3.6 kHz. Intersegment gaps were relatively constant and ranged from 356 ms to 578 ms (409±57, n = 13).

### Generation of Pseudo-target Sounds as Test Stimulus Set

**Figure 2:**
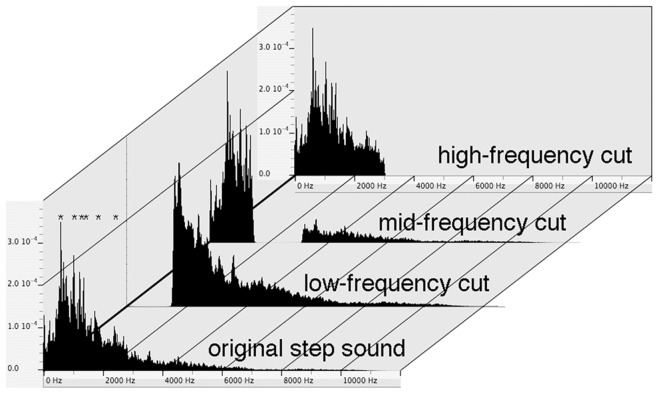
Power spectra of the footstep sound and its spectrally modified versions with different frequency bands removed. Power (relative) is on the y-axis, and spectrum (Hz) is on the x-axis. Overall power levels of the spectrally modified sounds are adjusted to be equal to that of the original target sound.

Pseudo-target (PsT) sounds were generated by digitally modifying the original T sound in the spectral and temporal dimensions on sound editing software (Fig. 1D). “Min” and “Maj” versions were generated by duplicating the 7 minor and 7 major segments of the 14-segment T sound, respectively. The “Int” version was generated by expanding all intersegment intervals of the T sound by a factor of 2.0. The “Ord” version was generated by randomizing the segment order of the T sound without changing the segment intervals. “LC” and “HC” versions were generated by eliminating the frequency range lower than 1.5 kHz (39 Hz transition width at the cut-off edge) and higher than 3.1 kHz (56 Hz transition width at the cut-off edge), respectively (Fig. 1D and Fig. 2). Similarly, the “MC” version of the T sound was generated by eliminating the mid-range frequencies between 1.5 kHz and 3.1 kHz. The entire spectral range of the T sound was empirically divided into 3 partitions so that human listeners could perceive unambiguous differences in sound quality among the 3 ranges. The relative energy ratio of the eliminated portions was 0.55∶ 0.26∶ 0.20 for the “LC”, “MC”, and “HC” versions, respectively. When played back to animals, the overall energy level (RMS unit) of these band-removed sounds was equalized to that of the original T sound. For the PsT sound “R”, the entire T sound was reversed in time (Fig. 1D). Finally, for the “sR” version, only the segment portions were locally time-reversed, with the segment order unchanged (Fig. 1D).

### Training Procedures

Instrumental conditioning with food rewards started on the day after the transfer of animals to their home cage. Training consisted of a one-week adapting stage, followed by 3 training stages lasting 2 weeks. Throughout these stages, animals were weighed daily and fed according to their body weight. During the adapting stage, the animals’ body weight was gradually decreased to 85%–90% of the weight measured on the day that they were moved to the laboratory, and thereafter, it was maintained or increased slightly each day. Water was freely accessible throughout the experiment. A newly designed training procedure was introduced to facilitate the stimulus-reward association based on natural social behavior: “*frequent conflict among cage-mates for access to food*” [18]. Two or 3 animals were caged as a group and trained together throughout the 1st and 2nd stages. They were individually trained in the next (3rd) stage, and then they were subjected to a recognition test separately on the next day. Detailed procedures for training guinea pigs are described in Text S1.

### Presentations of Stimulus Sets in the Training and Test Sessions

The pressure levels of the stimulus sounds that were played back from the speakers varied from sound to sound, but they all fell between 54 dB and 64 dB SPL on average (RMS). In each training session, playback of the stimulus set, including 1 T sound and 7 NT sounds of different types, was repeated 7 times, with randomization of the sound order in each set. Consequently, the overall training stimulus session included 56 sounds. Animals were fed at a fixed delay (reward delay, 4.2 s) after the offset of each T sound. To assess the animals’ recognition of the T sound, pseudo-target (PsT) sounds of 7 different types were interleaved into the training stimulus set, with each sound type occurring only once per set (i.e., 63 sounds per test stimulus session). Time intervals between the adjoining sounds were varied but were kept relatively constant, ranging from 47 s to 78 s (median 61 s), except the interval immediately after the T sound. This interval duration was roughly twice as long, ranging from 105 s to 130 s (median 117 s), allowing the animals to finish consuming the pellets before the next sound started. Sessions for the training and the recognition tests lasted approximately 70 and 80 min, respectively.

### Assessment of Animal's Behavioral Reactions and Data Analyses

**Figure 3:**
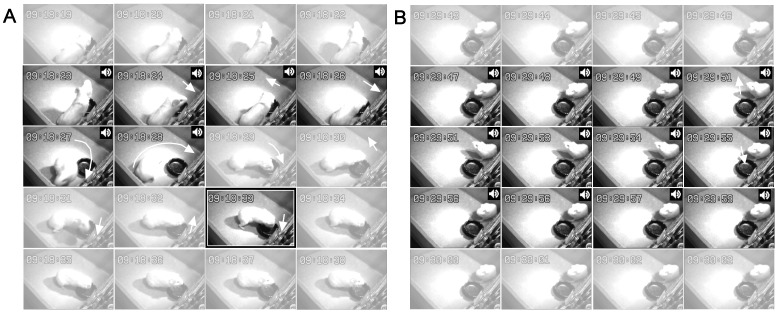
Sequences of video frames showing the typical behavioral responses to target and non-target sounds. Following the playback of the target footstep sound (**A**), a fully conditioned guinea pig displays circling locomotion and/or quick head swaying. Such reactions are not evoked by a non-target sound (**B**). Frames are taken approximately every 1 s. Sound-on periods are indicated by speaker symbols. Pellets are fed at the timing of the enclosed frame (**A**). Head motions are indicated by a set of white short arrows with the arrowhead pointing to the direction of movement. The track of the circling locomotion is depicted by a set of white curved arrows.

Behavior of the animal(s) was continuously monitored using 3 video cameras placed at different angles. The time window for assessing behavioral reactions started at the sound onset and ended at the feeding for the T sound stimulus or at the equivalent time for the NT and PsT sound stimuli (i.e., sound length +4.2 s). The behavioral reactions were inspected both on-line and off-line. If distinct circling and/or head-swaying behaviors (Fig. 3, also see Movie S1) were initiated within the time window, the animal's response was assessed as positive. The reactions were evoked soon after sound onset (3.0 s ±2.9, n = 81). The time windows of all stimulus sounds were long enough to prevent NT sounds shorter than the T sound from being scored as negative due to an insufficient time window. A distinction between spontaneous behaviors and positive reactions could be reliably made because the behavioral reactions were characterized by their abruptness, speed and repetitiveness, giving an impression of a “bustling” or “hurrying” state (Movie S1). Thus, either false negatives or false positives were extremely rare.

### Statistical Analyses

**Figure 4:**
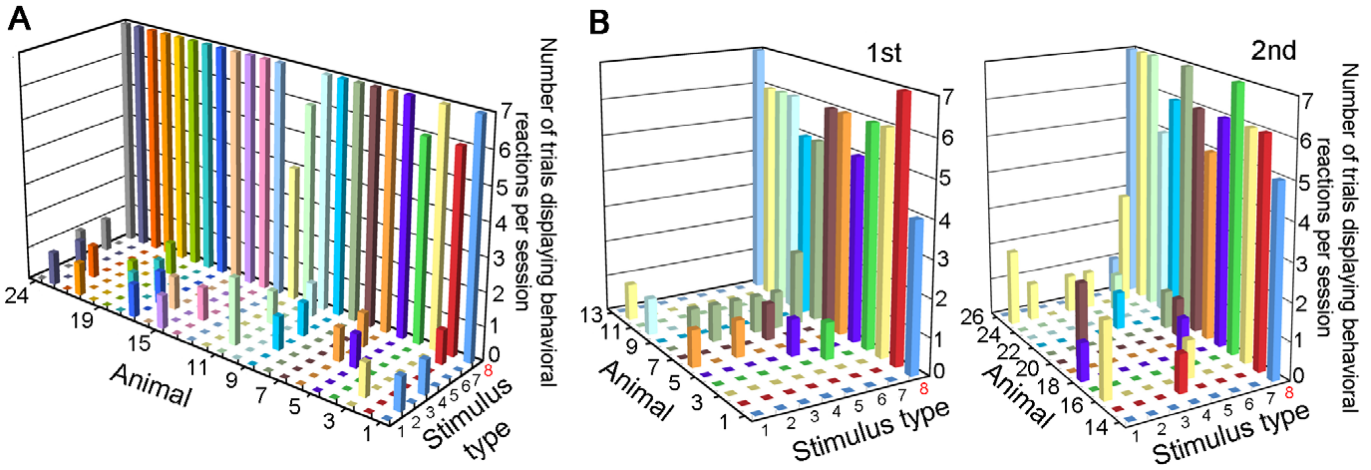
Graphs showing the number of trials displaying the behavioral reactions to target and non-target sounds. **A**. Responses to target (8) and non-target (1–7) sounds in the confirmation tests carried out as the last session of training. **B**. Responses to target and non-target sounds included in the recognition tests. Recognition tests were carried out one day after the confirmation test (*left*, 1st test; *right*, 2nd test). X-axis shows sound types: 1, tap water; 2, clapping hands; 3, hitting plastic carrier; 4, hitting metal plate; 5, scratching metal mesh; 6, jingling keys; 7, human vowels; and 8, footstep sound (target). Y-axis, animals tested. Note that each stimulus type is repeated 7 times per session; thus, the maximum number of positive trials is 7 (z-axis). p>0.5 for any pair between non-target sounds, while p<0.01 for any pair between target and non-target sounds (see text for statistical details).

**Figure 5:**
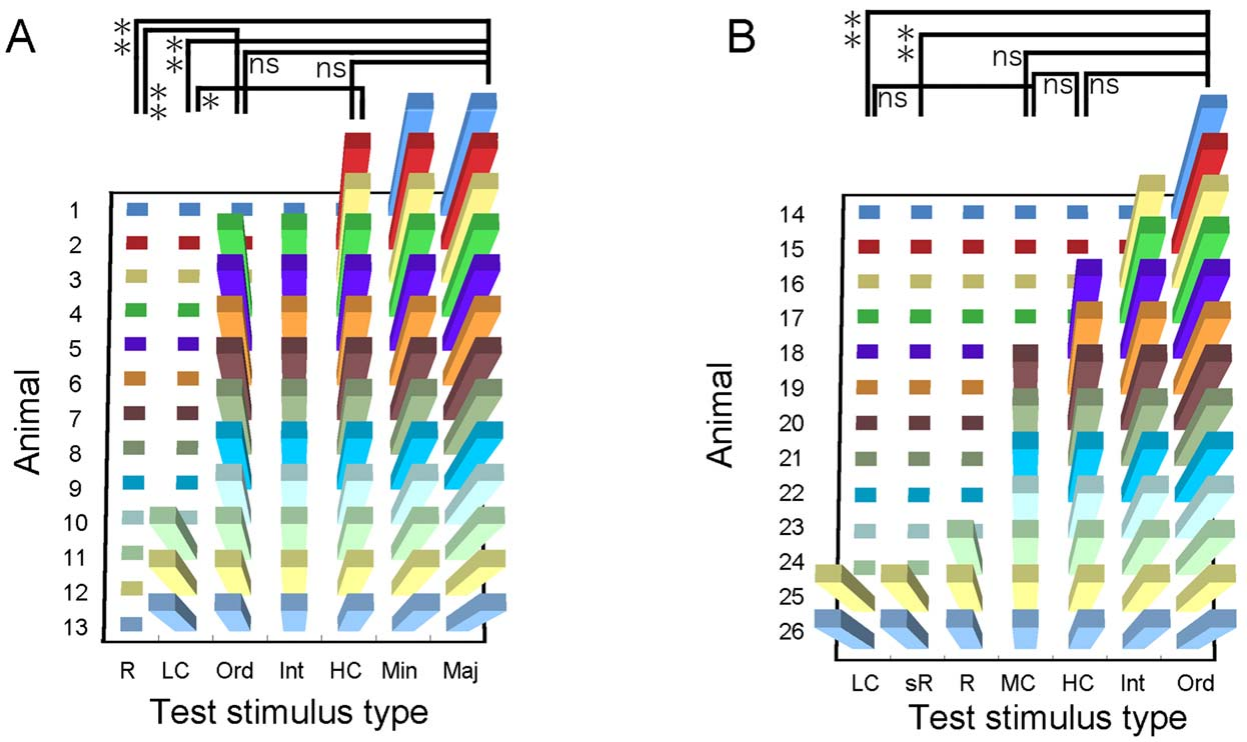
Graphs displaying the presence (bar) or absence (square) of the behavioral reactions to different pseudo-target sounds. Slightly different stimulus sets (x-axes) were prepared for 2 separate groups of animals (**A**, 1st test, n = 13; and **B**, 2nd test, n = 13) (y-axes). The pseudo-target sounds used in the recognition tests are shown on the x-axes. “Maj” and “Min”; modified step sounds with 7 major and 7 minor segments of the step sound doubled. “Int”; modified step sound with the intersegment intervals expanded. “Ord”; modified step sound with the segment-order shuffled. “LC”, “MC” and “HC”; modified step sounds with the low-, mid-, and high-frequency range removed from the step sound, respectively. “R” and “sR”; modified step sounds with the entire sound reversed in time and with individual segments locally time-reversed, respectively. *, p<0.05 and **, p<0.01 and n.s., not significant on a non-parametric statistical test (see text for statistical details).

Conditioning was confirmed in the final session of the last training stage (confirmation test). Different stimulus types (1 T and 7 NT) were ranked according to the response rates for each of the animals used, as displayed in Figure 4A (n = 24; 2 animals were excluded because of technical recording errors). These data were subjected to a non-parametric Friedman test for multiple comparisons, followed by a post hoc Nemny's test among the stimulus sound types. For the recognition test, different PsT stimulus types were assigned either a positive or negative sign in each animal according to whether the behavioral reactions were evoked or not (Fig. 5). These data were subjected to a non-parametric Cochran' Q test, followed by a post hoc McNemar test with Bonferroni corrections to selected pairs of the PsT sounds. Statistical comparisons among the T and NT sounds included in the recognition test sessions were also analyzed among the stimulus sound types in the same way as those used for the confirmation test (Fig. 4B).

## Results

### Behavioral Reactions to the T and NT Sounds after Conditioning

A total of 28 guinea pigs began training. Two of these animals became severely immobile soon after being moved from the animal facility and could not be conditioned with a food reinforcer in the routine training manner. These animals were excluded from the analysis. The remaining 26 animals advanced to the final stage in the 2-week training program and were subjected to a recognition test. The stimulus set used for the training consisted of 8 different sounds (1 T and 7 NT sounds), which were repeated 7 times per session with their order randomized in every set. Waveforms of the NT (no. 1–7) and T (no. 8) sounds, with a few segments of the T sound expanded in time and enlarged in amplitude for clarity, are shown in Fig. 1B. Once guinea pigs were conditioned to the T sound, distinctive and presumably innate behavioral reactions were reliably evoked after the T sound onset but prior to feeding (Fig. 3A). These behavioral reactions were characterized by two distinctive motions that could be easily differentiated from spontaneous movement, such as randomly approaching the food saucer and licking or sniffing at the food hopper. One of these reactions was an abruptly evoked circling behavior around the food saucer, with the diameter of the circle varying from trial to trial and from animal to animal (Fig. 3A, Movie S1). The other, more stably evoked reaction was head swaying (Fig. 3A, Movie S1). This movement was quickly repeated along the front-back and left-right axes while the animal kept its muzzle either within the food saucer or directed toward the feeding hopper. Animals typically ignored the NT sounds by staying stationary or by directing the head transiently towards a speaker without swaying the head (Fig. 3B).

The last session of the 2-week training period was used as the confirmation test. All animals showed behavioral reactions almost exclusively to the T sound, with very rare false alarm responses to NT sounds (Fig. 4A). In this 7-trial test session, the per-session average of the number of trials displaying positive responses to the T sound was 6.7 across all animals (n = 24, 2 animals excluded), while responses to the NT sounds were very low, ranging 0.08 to 0.29. A non-parametric Friedman test revealed that the behavioral reactions were more frequently evoked by the T sound than by any of the NT sounds (p<0.01 for all possible pairs between the T and NT sounds, whereas p>0.5 for all possible pairs between different NT sounds).

### Behavioral Reactions to the First Set of PsT Sounds

On the day following the confirmation test, the animals were tested in a single behavioral session composed of 7 different target-like (PsT) sounds in combination with a set of the stimulus sounds used in the confirmation test. Waveforms of the PsT test sounds are shown in Figure 1D.

Slightly different recognition tests were designed for 2 independent populations of animals. For the first group of 13 guinea pigs (Fig. 5A), behavioral reactions were evoked in response to the “Maj” (7 major segments of the T sound doubled) and “Min” (7 minor segments of the T sound doubled) versions of the T sound in all trials (13/13 animals). Behavioral reactions to the “HC” (high-frequency components removed from the T sound, Fig. 2), “Ord” (segment order randomized), and “Int” (intersegment intervals doubled; see “interval” in Movie S1) versions were evoked in most trials (12/13, 10/13 and 10/13, respectively). In contrast, reactions to the “LC” (T sound without low-frequency component, see “low-cut” in Movie S1) and “R” (entire T sound reversed in time) versions were rarely evoked (3/13 and 0/13, respectively). A non-parametric test showed that the “R” version was perceived differently from the “Maj” (p<0.01) and “Ord” (p<0.01) versions. Similarly, the “LC” version was perceived differently from the “HC” version (p<0.05) as well as from the “Maj” version (p<0.01).

The above results indicate that both spectral and temporal parameters are important for the recognition of non-harmonic natural sounds by guinea pigs. Low-frequency components are necessary for guinea pigs to recognize the T sound because the guinea pigs behaviorally distinguished between a sound containing such components (T sound) and a sound lacking them (“LC” version). In contrast, the animals rely less on high-frequency components because they responded similarly when the sound contained higher spectral components (T sound) and when it lacked them (“HC” version). Thus, low-frequency components, but not higher ones, are necessary for animals to extract the biological significance of the noise-like natural sound.

Temporal structures also contributed to the recognition of the noise-like natural sound because the “R” version, in which the entire T sound was reversed in time, was perceived to be different from the T sound (Fig. 5A and B). In this version, the overall order of the constituent segments was also changed. This apparently contradicted the findings that animals consistently showed a positive behavioral response to the other temporally modified PsT sounds in which either the order of the segments was changed (“Ord” version) or the overall rhythm was changed (“Int” version) (Fig. 5A and B). However, it should be noted that these 2 versions were modified only in terms of the overall envelope shape or coarse temporal structure, but the fine temporal structures embedded in the individual segments were not modified (or reversed in time). Therefore, it is possible that animals rely dominantly on fine but not coarse temporal structures.

### Behavioral Reactions to the Second Set of PsT Sounds

To verify the above assumption, a second recognition experiment was conducted with a separate group of animals (n = 13, Fig. 5B) in which the segment portions of the T sound were locally time-reversed without changing their overall order (segment reversed, “sR”, Fig. 1D). The behavioral reactions to the “sR” version were evoked in very few animals (2/13 animals) (Fig. 5B and “sR” in Movie S1). Similarly to the first type of recognition test, the “Ord” version as well as the “Int” and “HC” versions evoked reactions in most animals (13/13, 11/13 and 9/13, respectively), while the “R” and “LC” versions evoked reactions in very few animals (3/13 and 2/13). The same statistical test used in the first recognition test showed that the “sR” version was perceived differently from the “Ord” versions (p<0.01). These results suggest that our assumption that animals rely dominantly on fine temporal structures is correct.

The 2nd stimulus set also included another new PsT sound with the mid-range frequencies of the T sound deleted (“MC” version; Fig. 2). This PsT version was composed of the 2 separate spectral ranges that had been eliminated from the “LC” and “HC” versions. Behavioral results showed that the reactions to the “MC” version varied considerably among animals (see “middle-cut” in Movie S1 for a case of no reaction). The probability of displaying reactions to the “MC” version (7/13 animals) was between the probabilities of displaying reactions to the “LC” and “HC” versions (not significant for pairs between “MC” and “LC”, p>0.1; and between “MC” and “HC”, p>0.1; Fig. 5B).

### Behavioral Reactions to the T and NT Sounds Played Together with the PsT Sounds

The stimulus sets used for the recognition tests included both sounds used in the training session as well as the PsT test sounds. Behavioral responses to the T and NT sounds in the recognition tests were also assessed. The response pattern to the T and NT sounds was very similar to the confirmation tests (compare Fig. 4A with Fig. 4B). The same statistical analysis as that used for the confirmation tests showed p<0.01 for all possible pairs between the T and NT sounds and p>0.5 for all possible pairs between different NT sounds.

## Discussion

### Temporal Components as Cues in Noise-like Sound Discrimination

The T sound is a looming sound consisting of 14 short noise-like segments (each, on average, 110 ms long) corresponding to noises generated by individual step motions. These acoustic stimuli involve two temporal structures: one is the relatively fine temporal structure characterizing individual segments, and the other is the coarse temporal structure representing the overall envelope of the entire sequence of 14 segments. The guinea pigs that had consistently discriminated between the T and NT sounds did not discriminate the sounds with altered segment order (“Ord” version) or altered inter-segment intervals (“Int” version) from the T sound, which represented changed overall envelope structures or changes in sound rhythm. This result suggests that guinea pigs do not rely on the coarse temporal structure for the identification of noise-like natural sounds. These types of PsT sounds can be regarded as naturally occurring variations in human or animal steps that presumably belong to the same category as the object or event that might generate the T sound.

No obvious behavioral reactions were evoked to the reverse versions of the T sound, regardless of whether the reversal was local (“sR” version) or global (“R” version). Thus, it is likely that both reversed versions were recognized as different from the T sound. The response rates to these 2 stimuli were almost the same (2/13 vs. 3/13, p = 1.0, McNemar's test). This suggests that the entire envelope configuration of the sR sound, which is globally the same as the T sound, is not used as a cue for discrimination. These results again indicate that the guinea pigs are not sensitive to the overall envelope shape but are specifically sensitive to fine temporal structures embedded within individual segments.

Both the “R” and “sR” versions were derived from the T sound recorded in a standard experimental room, but not in an anechoic environment. The individual segments were therefore asymmetric in temporal structure. Individual segments exhibited an onset of a relatively sharp-rising phase with a gradually damping offset (Fig. 1B). The guinea pigs’ sensitivity to the reversed structures suggests that the envelope shape of the 110 ms-long segment(s), or the sequence of putative shorter sub-segments, is crucial to identification of the sound. For a given harmonic sound composed of a countable number of component frequencies, the phases and amplitudes of the component frequencies determine the sound’s envelope structure. In birds, it was recently shown that phase synchrony differences as small as tens of milliseconds in the component harmonics buried within syllables are critical cues for song recognition [11], [12]–[19]. Although the noise-like T sound used in the present study consisted of an uncountable number of component frequencies (Fig. 1C–9), it was composed of a countable number of “peaks” of local energy maxima (Fig. 2). If a set of energy peaks in a noise-like sound serves as a predominant determinant for its identity, similar to the formant structure of harmonic sounds, then it would be interesting to see whether the spectral position or relative phase of “peak” frequencies might also be a discrimination cue to guinea pigs.

The capacity to remember sequences of transient acoustic structures is critical to the recognition of auditory objects and is especially prominent in human words and speech comprehension. Our results showed that guinea pigs did not discriminate the “Ord” (segment sequence randomized) or “Int” (inter-segment gaps increased) versions from the T sound. There may be multiple interpretations of this poor discriminability. However, available data from various animal species [20]-[24] suggest that short-term memory for sounds extends up to several seconds, a range that can cover the inter-segment gaps of our multi-segment T sound. Assuming that guinea pigs also have this short-term memory, they could retain the temporal sequence of the individual segments. Despite this potential retention, they did not discriminate the temporally modified versions of the T sound from the original sound. Thus, they may ignore or not use the sequence cues of the segments; instead, they may simply extract enough information to initiate the behaviors from a single segment or a few segments. The observation that animals initiated behavioral reactions soon after stimulus presentation (i.e., approximately 3 s after T sound onset) supports this possibility. In this respect, birds are more capable of memorizing temporal structures because they rely on motif-sequences to recognize structured song signals [25]. Specific training paradigms in which the temporal interactions of short-term memories are assessed will be needed to fully elucidate the neural mechanisms that underlie the perception of the whole from parts of the temporal domain.

### Spectral Components as Cues in Noise-like Sound Discrimination

With regards to spectral composition, PsT sounds that lacked low-frequency components (“LC” version) were consistently discriminated from the T sound from which the PsT sounds originated. Various studies have demonstrated that animals depend on harmonic elements for behavioral initiation. For example, instinctual behaviors in mother-pup call communication are dependent on particular harmonic elements or the combination of elements in the calls [26]–[27]. The detection of acoustic signals emitted by offspring is known to cause particular behaviors of their mother; wriggling calls by rodent pups consistently cause their mother to initiate licking behavior, change suckling positions, or retrieve pups [28]–[29]. However, there are few studies focusing on mechanisms of how animals respond to and recognize biologically significant noise-like non-harmonic signals, despite the ubiquitous presence of these signals in the environment. The present results show that discrimination of a non-harmonic sound by guinea pigs depends on the frequency composition of the sound but may not share mechanisms with harmonic sound discrimination. The spectral range necessary for the discrimination of noise-like sounds likely requires a wider bandwidth because the animals did not differentiate behaviorally between the T sound and its spectrally modified version in which a very narrow spectral band was removed at the largest energy peak (1/6 octave at 650 Hz; 92% response rate in 12 trials with 3 animals; unpublished observation). This spectral dependency is different from harmonically structured sounds. The pitch perception of harmonic sounds is strongly dependent on a single component, the fundamental frequency. It is presumed that different frequency-based cues are used for the discrimination of harmonic and non-harmonic sounds. In this respect, the present results may help elucidate such frequency-based cues.

Frequency components other than the lower range also contributed to broadband sound discrimination in the current study. Pseudo-target sounds that lacked high-frequency components (“HC” version) were discriminated from the T sound in very few sessions (or animals), and those that lacked mid-frequency components (“MC” version) were discriminated in the majority of the sessions. Thus, the discrimination of the “MC” version from the T sound was inconsistent among animals, suggesting that the saliency of its discrimination resides between the “LC” version (almost full discrimination) and the “HC” version (almost null discrimination). This result implies that, compared to the “HC” version, the “MC” version lacks a certain factor that is necessary to categorize it with the T sound; moreover, compared to the “LC” version, the “MC” version lacks another factor that is required to discriminate it from the T sound. Furthermore, considering that the “MC” version contained the lower frequency components that were eliminated in the “LC” version and were thus indispensable for the T sound identification, the intermediate (but not-full) impact of the “MC” on discrimination suggests that the low-frequency component may not be “sufficient” for T sound identification. This conclusion implies that the relative energy distribution in the spectral dimension is the critical factor determining how much spectral components contribute to conditioning animals when broadband sounds are used as acoustic stimuli. In the current spectral modulation, the relative weight of the energy assigned to the spectrally eliminated portions was 0.55, 0.26 and 0.20 for the “LC”, “MC” and “HC” versions. The ranking corresponds to the discriminability of these 3 PsT sounds. This ranking may be comparable to the relative contribution of different spectral ranges for the intelligibility of noise-vocoded speech by humans [30]. Our data support the idea that the spectral composition is a major determinant in the recognition of non-harmonic broadband sounds.

### Comparison to the Perception of Harmonic Sounds

The mechanisms of recognition or perception of harmonic sounds, such as human speech sounds [30]–[31], animal calls [5] and birdsongs [11]–[12], [32]–[34], have been extensively studied. Recently, there has been increasing interest in the fine temporal structure of harmonic sounds in human speech intelligibility [35]–[36]. Findings indicating that birds attend to the local syllable structure of harmonic songs for learning [12]–[32] suggest a common mechanism between the voice quality discrimination of humans and the call discrimination of birds. In contrast, the recognition or perception of non-harmonic broadband signals, including environmental sounds, has been less systematically investigated. However, environmental sounds are closely related to the auditory “objects” from which the sounds are derived or generated [37]. Brain areas corresponding to the cortical representations of auditory objects have been explored in healthy [38] and brain-impaired [39] human subjects. In humans, the processing of environmental sounds related to hand-manipulated tools involves the activation of widespread cortical areas that are interconnected as a network and specifically activates areas linked to the dynamic motor actions responsible for the generation of those sounds [38]. Because environmental sounds are acoustically complex and derived from variable objects in continuously changing ambient situations, the brain areas for their perception must include unique networks [40] that are possibly widely distributed across the brain. The current guinea pig model could contribute to an analysis of neuronal responses participating in such networks.

### Conclusions

It seems premature to state that similar acoustic cues would be used for the recognition of other non-harmonic sounds. We used one particular sound, the sound of a human footstep, to represent non-harmonic natural sounds and modified it in the spectral and temporal dimensions on a relatively global scale. The modifications included the elimination of relatively wide ranges of frequency components and the disturbance of the overall timing of constituent segments of the multi-segment sound. It still remains possible that guinea pigs perceive sounds by relying on parameters on much finer scales than those used. For example, it is known that bird call recognition is disturbed by the modification of sub-syllable structures on a scale of milliseconds or tens of milliseconds [12]. This time scale is much smaller than the length of any segment of our multi-segment conditioning sound. Furthermore, with regard to the spectral structure, a frequency deviation of one higher harmonic component of a harmonic sound leads to the perception of inharmonicity in birds [11], suggesting that only a very subtle disturbance of spectral structure has a large impact on sound discrimination. Because such fine structures vary from sound to sound, evaluations on different spectrotemporal scales may be needed before we can conclude that the behavioral variations evoked by our test stimuli could be generalized to other non-harmonic sounds.

Answers to basic questions, such as how natural sounds are encoded or represented by neuronal networks, require good model systems in which sound perception can be assessed behaviorally as well as physiologically. The present study has clarified the basic acoustic cues that guinea pigs, used traditionally for studies of peripheral auditory mechanisms but rarely for auditory cortical mechanisms, use to discriminate among noise-like natural sounds. The guinea pig will provide an interesting mammalian model. Considering that the guinea pig has long been used as a model for cochlear implants [16], evaluation of its sound recognition would be a powerful tool to study functional restoration after cochlear implant placement and to understand central hearing mechanisms in mammals, including humans.

## Supporting Information

Text S1 **Detailed procedures of training guinea pigs are described in this protocol.**
(DOCX)

Movie S1 **Movie showing the behavioral reactions to target (T), non-target (NT), and pseudo-target (PsT) sounds.** The trials appear in the following order: (1) the “footstep sound” (T) with the behavioral response, (2) the “plastic carrier” sound (NT) with no response, (3) the “interval” modified version of the step sound (PsT) with a behavioral response, (4) a second “step sound” (T) with a behavioral response, (5) the “low-cut” version (PsT) with no response, (6) the “middle-cut” version with no response, (7) a third “step sound” (T) with a behavioral response, and (8) the “segment Reversed (sR) ” version (PsT) with no response.
(MP4)
