# Supplementary material for: Recognition of Non-Harmonic Natural Sounds by Small Mammals Using Competitive Training
Source: PLoS One. 2012 Dec 10;7(12):e51318. doi: 10.1371/journal.pone.0051318 (PMC3519678; doi:10.1371/journal.pone.0051318)
Supplement: Text S1 — Detailed procedures of training guinea pigs are described in this protocol. (DOCX) [file pone.0051318.s001.docx]

SUPPORTING INFORMATION

**Test S1. Details of the training procedures.**

**Text S1. Details of the training procedures.**

Pretraining Stage (adapting period). This stage was designed for three purposes, first for accustoming animals to a new environment, second for determining an adequate diet schedule, and third for associating target sound with reward in a naturalistic environment. Training was conducted mostly during the daytime, and the stage lasted maximally one week (excluding Sunday). Restricted diet started with fast on the day of transportation (day 1) from the university animal facility. Over the following few days, reduced amounts of pellets (RC4, Oriental Yeast Co. Ltd. or Guinea Pig Plus, Sanko, Japan) were fed to the animals kept in a group (on average 1.0 g per animal) several times per day. Further later, the number of feeding per day was increased to around 10 times, with a further reduced amount of food per feeding (on average, 0.5 g per animal). Whenever animals were fed, a feeder intentionally generated step sound while approaching the home cage. Special attention was paid to the total amount of food fed daily so that the body weights of individual animals gradually decreased to 85-90 % of the one measured on the day 1 by the last day of the stage. Water was freely accessible in all stages. Body weight of each animal was checked once or twice per day throughout the training.

Stage 1. This stage was designed for conditioning animals intensively to a digitized footstep sound in a new environment including measuring instruments placed within the sound-attenuated chamber. It lasted approximately 7 days. Two or 3 guinea pigs that had been kept in the same home cage were placed together in a training arena. As in the preceding stage, the feeder intentionally made step sounds while approaching the sound-attenuated chamber with its door kept open. A small amount of pellets (0.2-0.4 g per animal) was dropped manually into a saucer several second after he approached the front door of the chamber. Several days later, this manual feeding was replaced by a semi-automatic feeding using a custom-made pellet dispenser, together with a playback of the recorded footstep sound as the conditioning sound stimulus. Timing of the playback and feeding was controlled outside of the chamber by a Macintosh platform computer through a sound editing software (Amadeus Pro, HairerSoft, UK). Once the animals adapted themselves to this shift, without showing immobility, then the door of the sound-attenuated chamber was closed. The total number of feeding in this stage was about 20-30 times per day. After the daily training, animals were returned to their home cage and given supplementary food a few times using an auto-feeder. The amount of pellets was carefully controlled so that the body weight of animals increased slightly from the preceding day.

Stage 2. This stage was designed for training the animals to discriminate the footstep target (T) sound from other non-target (NT) sounds, and therefore only the T sound was reinforced with food. The amount of pellets fed as reward was on average 0.1 g per animal per feeding. As in the preceding stage, training was performed to the grouped animals. This stage lasted maximally 3-4 days. In one session, the stimulus set including 1 T sound and 7 NT sounds of different types (see Fig. 1) was repeated 7 times. On average, 4 sessions were applied per day. In this stage, the guinea pigs in a group confronted with a competitive state of a "first-come, first-served" basis, although the amount of food was adjusted so that at least one pellet was supplied to every animal in a group.

Stage 3. The animals that had been trained together in the preceding stage were individually conditioned to the T sound in otherwise the same training procedure as in the stage 2. The training lasted maximally 3-4 days. Two or 3 sessions per day were applied to each animal. As the confirmation test, the number of trials that showed the behavioral reactions (Fig. 2) was evaluated in the final session of this stage. No efforts were made to wake animals even when they fell asleep during the confirmation test.

Stage 4 (test session). On the following day, animals were individually subjected to single-session recognition tests once. In this test, on the basis of the induction of their behavioral reactions to the T-like sounds (see Fig. 1, it was assessed whether or not they perceived such sounds as the T sound. Prior to the start of the recognition test session, each animal was fed 3-4 times in association with the T sound playback to alleviate possible starvation.
